# GeneHarmony: A Knowledge-Based Tool for Biomarker Discovery in Disease: Sjögren’s Disease vs. Rheumatoid Arthritis and Systemic Lupus Erythematosus

**DOI:** 10.3390/ijms26136379

**Published:** 2025-07-02

**Authors:** Micaela F. Beckman, Adam Alexander, Jean-Luc C. Mougeot, Farah Bahrani Mougeot

**Affiliations:** 1 Translational Research Laboratories, Department of Oral Medicine/Oral & Maxillofacial Surgery and Cannon Research Center, Atrium Health Carolinas Medical Center, Charlotte, NC 28203, USA; micaela.beckman@atriumhealth.org (M.F.B.); aalexa78@outlook.com (A.A.); 2 Otolaryngology/Head and Neck Surgery, Wake Forest University School of Medicine, Winston-Salem, NC 27110, USA

**Keywords:** computational biology, autoimmune disease, Sjögren’s disease, Sjögren’s syndrome, data mining

## Abstract

Sjögren’s Disease (SjD), Rheumatoid Arthritis (RA), and Systemic Lupus Erythematosus (SLE) are autoimmune diseases with overlapping genetic features, yet the etiologies of these diseases are poorly understood. Using these rheumatic diseases as an example of proof of concept, our aim was to develop a tool that simplifies analysis of gene–disease associations applicable to any disease and to perform comparisons. This tool is meant to provide insights into associated gene symbols and gene expression data to identify candidate biomarkers in common among these diseases. The Diseasesv2.0 and GTExv8 databases were utilized for data collection, providing searchable disease names, affiliated gene symbols, confidence scores (ranging from 0 to 5, with 5 being the most confident), and gene expression across the panel of 54 tissue types present in GTExv8. Data infrastructure was established on a Postgres database using Plotlyv5.17.0 and Streamlitv1.27.2 Python packages. The resulting database was used to investigate the genetic associations among SjD, RA, and SLE, including confidence scores from 2.50 to 5.00. STRINGv12 analysis determined significant pathways (FDR < 0.05). Analysis using our tool revealed the following refined gene associations for each disease: SjD based on ‘Sjogren’ search term (*n* = 12 genes), RA (*n* = 231 genes), and SLE (*n* = 137 genes). We found seven genes in common, namely, *CD4*, *CD8A*, *IL6*, *IL17A*, *TNFS13B*, *TNF*, and *TRIM21*. With the exception of IL17A, these genes were expressed in tissue types known or suggested to be affected by SjD. STRINGv12 determined significant KEGG pathways involving interleukin signaling, cytokine signaling, and the immune system. We developed a tool that simplifies the data mining process, allowing users to search for diseases of interest and view common gene associations and gene expression. Some of the genes identified through our tool may be further explored to better understand SjD pathogenesis and systemic impact.

## 1. Introduction

Autoimmune diseases are complex disorders where the immune system mistakenly attacks the body’s own tissues [1]. Sjögren’s Syndrome (SS), recently renamed Sjögren’s Disease (SjD), Rheumatoid Arthritis (RA), and Systemic Lupus Erythematosus (SLE) share overlapping clinical features and complicated diagnoses [2]. Furthermore, our understanding of the underlying mechanisms of these diseases is lacking. Genetic and/or microbial biomarkers could provide insights into disease pathogenesis, aid in early diagnosis, and guide the development of targeted therapies. Recent advances in bioinformatics and genomics offer new ways to explore the genetic basis of diseases. Integrating gene–disease association data with gene expression profiles could reveal novel biomarkers and therapeutic targets. However, the vast amount of data and the specialized knowledge required for analysis still face significant challenges.

To address this, we developed GeneHarmony, a knowledge-based tool to simplify gene–disease associations and gene expression in various tissue types. GeneHarmony leverages a database infrastructure incorporating data from sources like the Diseasesv2.0 database and the Genotype-Tissue Expression (GTExv8) database [3,4,5]. This tool aims to identify candidate biomarkers and serve as a versatile platform for the study of multiple diseases with overlapping or similar pathogenesis. By streamlining data mining and making complex datasets accessible, further insights into the mechanisms of diseases and similar conditions may be discovered, thereby promoting diagnostic and therapeutic development. GeneHarmony provides information obtained from publicly available resources and is available for download at https://www.github.com/mbeckm01/GeneHarmony.

## 2. Results

### Data Usage and Application Results

**Figure 1:**
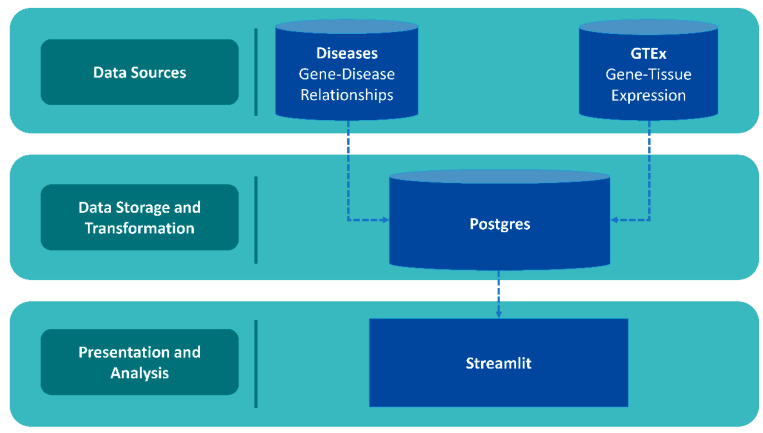
Entity-relationship diagram of GeneHarmony. Legend. Data was collected from the Diseasesv2.0 database applying the Amyloidoses Collection, MedlinePlus, Target Illumination GWAS Analytics (TIGA), UniProtKB-KW, and the Jensen Lab text mining sources for gene–disease associations and the Gene Tissue Expressionv8 (GTExv8) database for gene expression data. Data was utilized through PostgreSQLv16 with front-end development utilizing Streamlitv1.27.2 with Plotlyv5.17.0.

Data and accompanying scripts can be downloaded from the GeneHarmonyv1.0 GitHub page (http://www.github.com/mbeckm01/GeneHarmony). There are 40,288 unique diseases included in GeneHarmony for analysis from Diseasesv2.0. GTExv8 data contains gene expression values of 56,200 Ensembl gene ID’s for 54 tissue types (excluding normal immune cells). Figure 1 shows the entity-relationship diagram of GeneHarmony.

**Figure 2:**
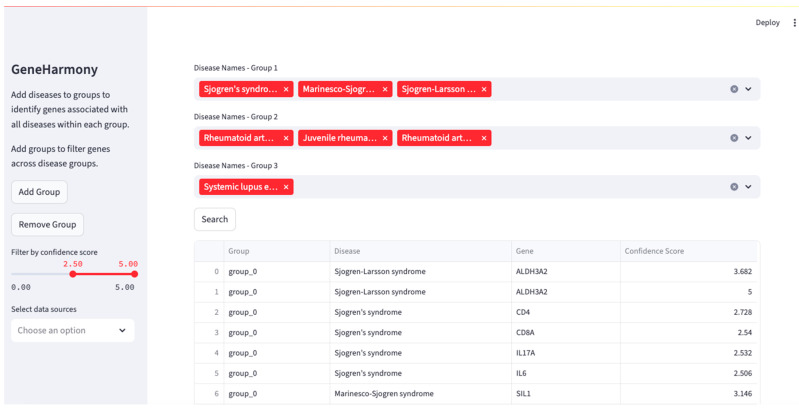
GeneHarmony case study investigating gene interactions between Sjögren’s Syndrome (i.e., Sjögren’s Disease), Rheumatoid Arthritis, and Systemic Lupus Erythematosus. Legend. Using GeneHarmony, Sjögren’s Syndrome (i.e., Sjögren’s Disease), Sjögren–Larsson Syndrome, and Marinesco–Sjögren Syndrome were selected for the first disease group. The second group focused on Rheumatoid Arthritis, including ‘Rheumatoid Arthritis’, ‘Juvenile Rheumatoid Arthritis’, and ‘Rheumatoid Arthritis Interstitial Lung Disease’. The query for the third group included only ‘Systemic Lupus Erythematosus’. All available gene–disease data sources were selected and the gene–disease confidence scores were set to span a range of 2.50 to 5.00.

A screenshot of the GeneHarmony-selected disease groups is presented in Figure 2. Initial query results revealed a significant number of genes associated with each disease group: 2000 genes for the first group, 6837 for the second, and 5453 for the third. Among these, 1421 genes were identified as common across all selected disease groups. Seeking to refine these results, we adjusted the gene–disease confidence score filter to a range from 2.50 to 5.00. This adjustment yielded a smaller set of genes: 12 for the first disease group, 231 for the second, and 137 for the third. The associated genes, pre- and post-filtering, are available in Supplemental File S1. Further, sensitivity testing of multiple confidence score ranges with the number of genes in common are presented in Supplemental Table S1. Seven genes were found to be common across all three disease groups after applying the refined filter. A Venn diagram showing the overlapping output can be found in Supplemental Figure S1. The resulting genes in common were CD4 molecule (CD4), CD8 subunit alpha (CD8A), interleukin 17A (IL17A), interleukin 6 (IL6), tumor necrosis factor (TNF), TNF superfamily member 13b (TNFSF13B), and tripartite motif containing 21 (TRIM21).

**Figure 3:**
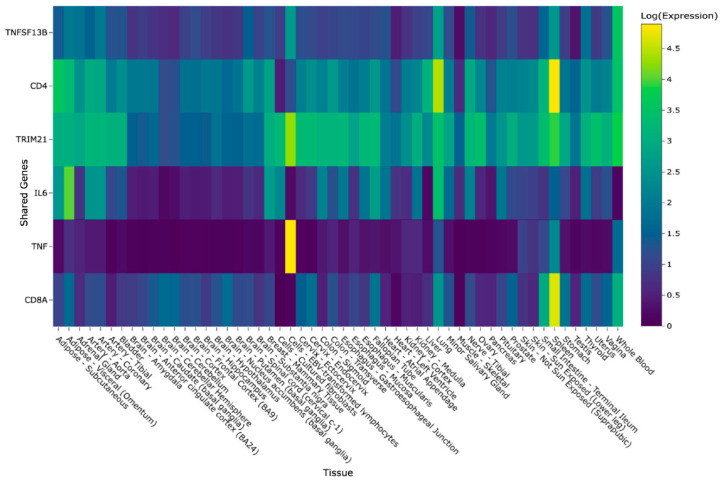
GeneHarmony case study heatmap showing intersecting genes within Sjögren’s Syndrome (i.e., Sjögren’s Disease), Rheumatoid Arthritis, and Systemic Lupus Erythematosus. Legend. Gene-tissue expression data was retrieved from the Gene Tissue Expression (GTExv8) database using normalized (N = log(N) + 1) transcripts per million (TPM) values. A screenshot from GeneHarmony showing a heatmap of the intersecting genes (*n* = 6) of three disease groups: (i) Sjögren’s Syndrome (i.e., Sjögren’s Disease), (ii) Rheumatoid Arthrtitis, and (iii) Systemic Lupus Erythematosus at a confidence score range from 2.00 to 5.00. IL17A, known to be expressed by Th17 cells and at a lower level by CD4^+^ regulatory rTh17 cells, was not expressed in any of the 54 tissue types presented in heatmap and is not shown.

Further interaction with the gene expression heatmap provided insights into the expression levels of the above-mentioned genes in various tissues. Notably, high expression levels were observed in log-adjusted TPM for the following tissues and genes: EBV-transformed lymphocyte tissues for TNF, spleen tissues for *CD8A* and *CD4*, and lung tissues for *CD4*. The heatmap presented within GeneHarmony is presented in Figure 3. IL17A, known to be expressed by Th17 cells and at a lower level by CD4^+^ regulatory rTh17 cells, was not expressed in any of the 54 tissue types presented in the heatmap [6].

The Search Tool for Retrieval of Interacting Genes (STRINGv12) analysis at a medium confidence resulted in all genes in common having protein–protein interactions. Additionally, 41 Kyoto Encyclopedia of Genes and Genomes (KEGG) pathways were determined as significant. Significant pathways included Antifolate resistance (hsa01523; FDR = 0.0017), Graft-versus-Host Disease (hsa05332; FDR= 0.0021), Rheumatoid Arthritis (hsa05323; FDR = 4.13 × 10^−6^), Hematopoietic cell lineage (hsa04640; FDR = 4.13 × 10^−6^), Yersinia infection (hsa05135; FDR = 4.92 × 10^−6^), and Systemic Lupus Erythematosus (hsa05322; FDR = 0.0082). The pathways for Rheumatoid Arthritis, Hematopoietic cell lineage, and Yersinia infection resulted in 4 of the 12 genes being included in the gene set. The gene TNF was present in 17 of 20 (85%) of the top KEGG pathways, while IL6 was present in 16 of the 20 (80%). The top 20 pathways are presented in Table 1.

## 3. Discussion

This case study demonstrates the power of GeneHarmony to facilitate complex analyses of gene–disease associations, allowing for a deeper understanding of the genetic overlaps in chronic diseases. Here, we were able to identify important genes in common between Sjögren’s Disease, Rheumatoid Arthritis, and Systemic Lupus Erythematosus, as well as their gene expression values in multiple tissue types. Furthermore, we showed how GeneHarmony can be used in downstream analysis to identify significant pathways related to genes in common. Through the application of specific filters and the analysis of gene expression data, we were able to highlight genes and pathways of interest that may play crucial roles in the pathology of these autoimmune diseases.

We were able to determine seven genes in common between the three disease groups, leading to downstream analysis confirming that the genes involved are related to the KEGG pathways of ‘Rheumatoid Arthritis’ and ‘Systemic Lupus Erythematosus’. Using GeneHarmony, we were able to identify TNF (*alias* TNF-α) and IL6 as important genes associated with all three disease groups, which might provide further insight into SjD development of autoimmunity systemically. The genes TNF and IL6 were the most important among all significant pathways being present in 85% and 80% of the top 20 significant KEGG pathways, respectively.

TNF may have conflicting roles in the immune system, having the ability to induce inflammatory processes as well as fight infections and provide immunity against pathogens [7]. TNF has been shown to play a role in the pathogenesis of SjD using mice models but its role in the immune system is complex and not fully understood [8]. In SjD, targeting TNF-α has been shown to be ineffective, resulting in continued dysregulated cytokine levels and even increased TNF-α levels after treatment [9]. In RA, TNF activates synovial fibroblasts causing cathepsin and MMP overproduction, resulting in the breakdown of collagen, cartilage, and bones [7]. There are several available TNF inhibitors that have proved to be effective in the reduction in disease progression in patients with RA [10]. A study investigating the role of TNF-α in SLE suggested that anti-autoimmune effects could lead to autoantibodies production and the stimulation of pro-inflammatory mechanisms in SLE development [11]. It has also been suggested that TNF-a inhibitors block access to regulatory TNFR2 and the pro-apoptotic TNFR1 receptors, resulting in deregulated signaling function of TNFR2 [12]. Due to this and the possibility of increased risk of infection, drug-induced Lupus Erythematosus, and possible increased risk of cancer, many healthcare professionals avoid using anti-TNF-α therapies as treatment for SLE [13,14,15].

A study by Grisius et al. found elevated concentrations of IL6 in serum of patients with SjD compared to healthy controls [16]. IL6 functions in the production of Th17 cells (expressing IL17A) and can activate local B cells. In SjD, this mechanism can lead to the release of more IL6 [17]. Like TNF-α, IL6 has diverse functions in the immune system and has recently become a prospective target for treatment of the disease. Targeting IL6 may disrupt its protective effects while failing to address the multi-inflammatory pathways involved in the development of SjD and SLE [18]. Additionally, IL6 is not seen as the primary driver of inflammation in patients with SjD or SLE. Instead, SjD and SLE are characterized by infiltration of T cells into tissues, creating a positive feedback loop through the production of pro-inflammatory cytokines through the induction of B cell activation [19,20]. Moreover, the ability of Th17 cells to change their characteristics and functions based on their microenvironment proves their status as challenging drug targets to alleviate symptoms and slow down the progression of autoimmune diseases, warranting a broader treatment strategy [21]. Drugs such as rituximab, tocilizumab, and bortezomib have conflicting results in the treatment of SjD, demonstrating inconsistencies, clinical improvements, or lack of systemic involvement improvement in IL6 and Th17/ IL17 [17,22,23,24,25].

This tool allowed us to see genes in common and determine possible future diagnostic and/or drug targets that have not previously been investigated for their effectiveness in the treatment of these three diseases. For example, TRIM21 was implicated in all three diseases but we were unable to identify any studies using drugs targeting TRIM21, such as vilazodone, in the treatment of SjD, RA, or SLE despite research suggesting it may lead to reduced pro-inflammatory gene expression and immune modulation [26]. Additionally, CD4 and CD8A are crucial components of the immune system, playing a vital role in T cell activation and function. The drug ibalizumab works to target CD4, binding to the CD4 receptor that prevents HIV from binding to CXCR4 or CCR5. CXCR4 has been shown to have increased expression in SjD patients, causing increased migration of immune cells into lacrimal glands [27]. Moreover, CXCR4 has been shown to be highly expressed in the serum and joint synovial fluid of patients with RA and upregulated in circulating B cells in patients with active SLE, promoting infiltration into renal tissues [28,29]. However, no studies have investigated ibalizumab for its efficacy in these diseases.

Currently, this tool does not consider sex-specific gene expression patterns and their contributions to diseases. In rheumatic diseases such as SjD, RA, and SLE, there is a known disparity in female-to-male incidence. Recently, global studies have placed the female-to-male ratio at a 14:1 ratio for SjD, a 3:1 ratio for RA, and a 4–14:1 ratio for SLE, depending on ethnicity [30,31,32]. More recent studies have highlighted the relationship between X chromosome presence and risk of developing autoimmune diseases [32,33]. These studies revealed women with Klinefelter (XXY) or Triple X (XXX) syndromes and men with Klinefelter syndrome (XXY) have similar risks of autoimmune diseases. However, it has been determined that women with Turner’s syndrome (XO) experience a decreased risk [32,34,35,36]. A gene found on the X chromosome, *CXorf21*, was found to be upregulated at the mRNA and protein level in women’s vs. men’s monocytes and overexpressed when under the effects of lipopolysaccharide (LPS) immunological challenge [37,38]. Further, the X-linked gene CXCR3, belonging to the same gene family as the previously mentioned CXCR4, has been implicated for its role in autoimmunity through its ability to direct the migration of T cells to sites of inflammation or injury [32,39]. Additionally, as women age, these genes have been shown evade X-chromosome inactivation, causing a dosage-dependent increase in gene expression and possibly contributing to the development of autoimmune diseases [40].

Imminent updates to GeneHarmony include incorporation of sex-specific gene expression data via the sex-associated gene database (SAGD) [41] to account for sex-based genetic factors and their contributions to diseases such as SjD, RA, and SLE. We also hope to incorporate GTExv8 filtering based on tissue type and expression quantitative trait loci (eQTL) data to identify common genetic variations across diseases of interest and to give insight into mutual tissue specificity and possible regulatory mechanisms. Furthermore, we hope to incorporate microbe set enrichment analysis (MSEA) data to explore the potential impact of LPS-producing bacteria on human gene expression in complex diseases, as well as data from the Human Microbiome Project to examine bacteria–tissue interactions, among other pertinent datasets [42,43]. This analysis has also highlighted the future need for experimental studies to confirm findings and standardized scoring methods to determine the relationships between genes and diseases/phenotypes from experimental, knowledge-based, and/or text mining databases.

In conclusion, while necessitating further development in integrating omics data to investigate rheumatic autoimmune diseases, GeneHarmony provides a valuable and user-friendly initial proof of concept data mining tool. 

## 4. Methods

### 4.1. Database Architecture and Data Collection

Our selection criteria for integrating data sources emphasize databases with a consistent history of updates, ensuring that our tool remains relevant and adapts to evolving data sources. Diseasesv2.0 was chosen for its gene–disease association data, weekly update schedule, comprehensive analysis from multiple sources, and its methodology for ranking the strength of gene–disease relationships [3]. Full-text ranking was taken into consideration using the scoring method calculated as confidence scores from 0 to 5 [3,5]. There is a reward mechanism built in for genes and diseases mentioned together, and there are penalties for genes and diseases being mentioned in conjunction with other diseases or genes [3,5]. Namely, confidence scores are used to determine how strongly a gene is linked to a disease using text mining. First, the number of times a gene and disease are mentioned together in an abstract or a sentence is determined. Next, a score is calculated in which the score increases if a gene and disease are mentioned frequently together but decreases if the gene is mentioned with many diseases or other genes. Scores are then normalized by being converted to a z-score to reduce background noise and refined into a confidence score from 0.0 to 5.0 to account for automatic text mining never being as reliable as a human manually determining annotations and associations [3,5].

The GTExv8, offering gene–tissue expression data from RNA-seq experiments, is similarly updated regularly, contributing to the continued improvement in our tool [4]. For visualization, gene–tissue expression data is normalized by placing it in a matrix and applying an adjustment [N = log(*n* + 1)] to the transcripts per million (TPM) values, creating a heatmap.

The data storage infrastructure, database, and schema were established using PostgreSQLv16 [44]. PostgreSQL is a free and open-source database, also known for its capability to integrate full-text indexing, essential for potential future machine learning applications [44].

For front-end development and user interaction, we used Streamlitv1.27.2 [45]. Despite the availability of many robust web application frameworks, we chose Streamlitv1.27.2 for its ability to quickly integrate data visualizations from various libraries commonly used in bioinformatics. This tool is easy to use by bioinformaticians without the need for extensive web application development experience. We implemented Streamlitv1.27.2 data frames for displaying query results, while matplotlibv3.10 and Plotlyv5.17.0 visualizations were used for the graphical representation of common gene counts and gene expression data, respectively [45,46,47].

### 4.2. Download and Usage

GeneHarmony requires the installation of miniconda, pip, psycopg2v2.9.10, and Streamlitv1.27.2, as well as matplotlibv3.10.3, pandasv2.3.0, numpyv2.3.1, kaleidov1.0.0, and plotlyv5.17.0 pythonv4.3.0 packages [45,46,47,48,49,50,51,52]. Furthermore, PostgreSQL is a pre-requisite for GeneHarmony. All database files can be downloaded from OneDrive. The ‘create_disease_names.sql’ script within the ‘Streamlit ETL directory’ is used to create a streamlined table for the Streamlitv1.27.2 application while ‘modify_gtex_tpm.py’ will set up all gene expression data for use. Streamlitv1.27.2 can be started using the following command: ‘Streamlit run streamlit.py’. From here, a browser should appear, and the local IP address should appear in the terminal after running the command. The initial setup does not require any user data.

User queries are initiated by selecting a disease name from a textbox. Multiple disease names can be selected for related diseases, allowing the identification of genes in common between similarly named diseases. Additional disease groups can be added to the query to identify genes intersecting between the disease groups. When the query is executed, genes associated with each disease group are returned, along with their intersection, which is then visualized via a Venn diagram and a heatmap. Users can further refine the results using filters such as the strength of the gene–disease relationship and the source of the gene–disease association.

### 4.3. Application: Utilizing GeneHarmony for Cross-Disease Gene Association Analysis

In this case study, we employed GeneHarmony to investigate the genetic associations among Sjögren’s Syndrome, Rheumatoid Arthritis, and Systemic Lupus Erythematosus. The exploration began by entering the term “Sjogren” into the search field, which auto-populated with three related diseases: Sjögren’s Syndrome, Sjögren–Larsson Syndrome, and Marinesco–Sjögren Syndrome, all of which were selected for the first disease group. The second group focused on Rheumatoid Arthritis, including ‘Rheumatoid Arthritis’, ‘Juvenile Rheumatoid Arthritis’, and ‘Rheumatoid Arthritis Interstitial Lung Disease’. The query for the third group included only ‘Systemic Lupus Erythematosus’.

The analysis proceeded without the application of any filters. All available gene–disease data sources were selected, encompassing AmyCo, MedlinePlus, Target Illumination GWAS analytics (TIGA), Jensen lab texting mining sources, and UniProtKB-KW [53,54,55,56]. The gene–disease confidence score was set to span the full range from 0.00 to 5.00. Results were then refined to only include genes scoring within the top 50% of possible confidence scores between 2.50 and 5.00 to limit false positives. Genes in common were then used for STRINGv12 analysis to determine any possible protein–protein interactions using the STRINGv12 database (www.string-db.org) at a medium confidence (0.400) [57]. A Venn diagram was used to visualize the counts of overlapping genes within each disease group. A heatmap was created within GeneHarmony to visualize gene expression of the overlapping genes in 54 tissue types.

## Figures and Tables

**Table 1:** Significant KEGG pathways.

KEGG Pathway ^a^	Pathway ID ^b^	Total # Genes ^c^	# Genes Included in Network ^d^	Genes Included from Analysis in Network ^e^	FDR ^f^
Antifolate resistance	hsa01523	31	2	IL6; TNF	0.0017
Graft-versus-host disease	hsa05332	36	2	IL6; TNF	0.0021
African trypanosomiasis	hsa05143	36	2	IL6; TNF	0.0021
Primary immunodeficiency	hsa05340	37	2	CD4; CD8A	0.0021
Inflammatory bowel disease	hsa05321	59	3	IL6; IL17A; TNF	6.91 × 10^−5^
Rheumatoid arthritis	hsa05323	83	4	IL6; IL17A; TNF; TNFSF13B	4.13 × 10^−6^
Antigen processing and presentation	hsa04612	64	3	CD4; CD8A; TNF	7.29 × 10^−5^
Intestinal immune network for IgA production	hsa04672	43	2	IL6; TNFSF13B	0.0024
Hematopoietic cell lineage	hsa04640	90	4	CD4; CD8A; IL6; TNF	4.13 × 10^−6^
Malaria	hsa05144	46	2	IL6; TNF	0.0025
Legionellosis	hsa05134	55	2	IL6; TNF	0.0034
IL-17 signaling pathway	hsa04657	91	3	IL6; IL17A; TNF	0.00017
Yersinia infection	hsa05135	124	4	CD4; CD8A; IL6; TNF	4.92 × 10^−6^
T cell receptor signaling pathway	hsa04660	100	3	CD4; CD8A; TNF	0.00019
Th17 cell differentiation	hsa04659	99	3	CD4; IL6; IL17A	0.00019
Pertussis	hsa05133	73	2	IL6; TNF	0.0055
Hypertrophic cardiomyopathy	hsa05410	88	2	IL6; TNF	0.0075
Systemic lupus erythematosus	hsa05322	94	2	TNF; TRIM21	0.0082
AGE-RAGE signaling pathway in diabetic complications	hsa04933	96	2	IL6; TNF	0.0082
Viral protein interaction with cytokine and cytokine receptor	hsa04061	96	2	IL6; TNF	0.0082

^a^ Top 20 Kyoto Encyclopedia of Genes and Genomes (KEGG) Pathways from the Search Tool for the Retrieval of Interacting Genes (STRINGv12) analysis of 12 genes in common between Sjögren’s Syndrome (i.e., Sjögren’s Disease), Rheumatoid Arthritis, and Systemic Lupus Erythematosus. ^b^ KEGG pathway identification. ^c^ Total number of genes included in the KEGG pathway. ^d^ Total number of genes included out of the 12 genes in common. ^e^ Entrez gene symbols of genes included in KEGG pathway. ^f^ False discovery rate.

## Data Availability

All supporting files and documentation for the download and use of GeneHarmony can be found on our lab’s Github page (www.github.com/mbeckm01/GeneHarmony).

## Supplementary Materials

The following supporting information can be downloaded at: https://www.mdpi.com/article/10.3390/ijms26136379/s1.
