# Supplementary material for: GeneHarmony: A Knowledge-Based Tool for Biomarker Discovery in Disease: Sjögren’s Disease vs. Rheumatoid Arthritis and Systemic Lupus Erythematosus
Source: Int J Mol Sci. 2025 Jul 2;26(13):6379. doi: 10.3390/ijms26136379 (PMC12250006; doi:10.3390/ijms26136379)
Supplement: Supplementary file 1 [file ijms-26-06379-s001.zip › Supplemental Figure 1. Venn Diagram of overlapping genes 06 4 25docx.pdf]

Supplemental Figure 1. GeneHarmony case study venn diagram showing intersecting genes within Sjögren's Syndrome (i.e. Sjögren's Disease), Rheumatoid Arthrits, and Systemic Lupus Erythematosus.

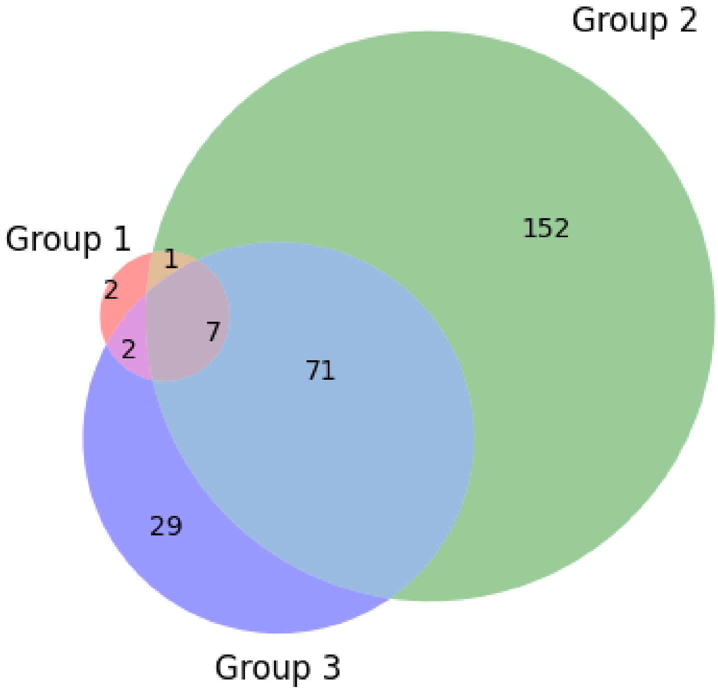

Legend. Venn diagram showing the number of intersecting genes between Sjögren's Syndrome (i.e. Sjögren's Disease; n=12 genes), Rheumatoid Arthritis (n=231 genes), and Systemic Lupus Erythematosus (n=137 genes) created using GeneHarmony, filtering using a confidence score range of 2.5 to 5.0. Group 1 (Sjögren's Disease) and Group 2 (Rheumatoid Arthritis) consisted of eight overlapping genes, Group 1 and Group 3 (Systemic Lupus Erythematosus) consisted of 9 overlapping genes while Group 2 and Group 3 consisted of 71 overlapping genes. Overall, there were seven genes in common among the three disease groups.
