# Supplementary material for: GeneHarmony: A Knowledge-Based Tool for Biomarker Discovery in Disease: Sjögren’s Disease vs. Rheumatoid Arthritis and Systemic Lupus Erythematosus
Source: Int J Mol Sci. 2025 Jul 2;26(13):6379. doi: 10.3390/ijms26136379 (PMC12250006; doi:10.3390/ijms26136379)
Supplement: Supplementary file 1 [file ijms-26-06379-s001.zip › Supplemental Table 1. Sensitivity Testing 06 24 25.pdf]

Supplemental Table 1. Sensitivity testing for confidence score ranges

**Confidence scores[a]**	**Gene count[b]**
0.0-1.0	78
0.0-2.0	1058
0.0-3.0	1400
0.0-4.0	1421
0.0-5.0	1421
1.0-2.0	229
1.0-3.0	546
1.0-4.0	568
1.0-5.0	568
2.0-3.0	23
2.0-4.0	47
2.0-5.0	47
**2.5-5.0**	**12**
3.0-4.0	0
3.0-5.0	0
4.0-5.0	0

Footnote. Sensitivity testing was completed using various confidence score ranges for the three disease groups searching for genes in common among the three groups: i) Sjögren's Syndrome, Sjögren-Larsson Syndrome, and Marinesco-Sjögren Syndrome, ii) Rheumatoid Arthritis, Juvenile Rheumatoid Arthritis and Rheumatoid Arthritis Interstitial Lung Disease and iii) Systemic Lupus Erythematosus.

[a]Confidence score ranges used for sensitivity testing

[b]Number of unique genes returned by GeneHarmony per confidence score range.

Note: the confidence score range shown in bold was the selected range for analysis in this study.
